# Prevalence and correlates of DSM-IV and DSM-5 Intermittent Explosive Disorder amongst Myanmar refugees living in Malaysia: a population-based study

**DOI:** 10.1017/S2045796022000257

**Published:** 2022-08-15

**Authors:** Alvin Kuowei Tay, Mohammed Mohsin, Susan Rees, Derrick Silove

**Affiliations:** 1 The Discipline of Psychiatry and Mental Health, School of Clinical Medicine, UNSW Medicine, Sydney, Australia; 2 Mental Health Research Unit, Liverpool Hospital, Sydney, New South Wales, Australia; 3 Department of Psychiatry, Phoenix Australia – Centre for Posttraumatic Mental Health, The University of Melbourne, Carlton, VIC, Australia

**Keywords:** Epidemiology, impulse control disorders not listed elsewhere, PTSD, risk factors, stressful life events

## Abstract

**Aims:**

We investigate the prevalence and risk factor profiles of Intermittent Explosive Disorder (IED) and comparison between two diagnostic measures for IED in a large population-based study of three ethnic groups of refugees (Chin, Kachin and Rohingya) from Myanmar resettled in Malaysia.

**Methods:**

Trained field personnel interviewed in total 2058 refugees, applying a clustered, probabilistic, proportional-to-size sampling framework and using the DSM-IV and DSM-5 criteria to diagnose IED. We used descriptive and bivariate analyses to explore associations of IED (using DSM IV or DMS 5) with ethnic group membership, sociodemographic characteristics and exposure to premigration traumatic events (TEs) and postmigration living difficulties (PMLDs). We also examined associations of IED with other common mental disorders (CMDs) (depression, anxiety and posttraumatic stress disorder) and with domains of functional impairment. Finally, we compared whether IED measured using DSM IV or DSM 5 generated the same or different prevalence.

**Results:**

For the whole sample (*n* = 2058), the 12-month prevalence of DSM-IV IED was 5.9% (*n* = 122) and for DSM-5, 3.4% (*n* = 71). Across the three ethnic groups, 12-month DSM-5 IED prevalence was 2.1% (Chin), 2.9% (Rohingya) and 8.0% (Kachin), whereas DSM-IV defined IED prevalence was 3.2% (Chin), 7% (Rohingya) and 9.2% (Kachin). Being single, and exposure to greater premigration TEs and PMLDs were each associated with IED. Over 80% of persons with IED recorded one or more comorbid CMDs. Persons with IED also showed greater levels of functional impairment compared with those without IED.

**Conclusions:**

The pooled IED prevalence was higher than global norms but there was substantial variation in prevalence across the three study groups.

## Introduction

Intermittent Explosive Disorder (IED) is a mental disorder characterised by impulsive and aggressive outbursts that are disproportionate to external provocation, although the reaction pattern can vary in severity across individuals (American Psychiatric Association, 2013; Scott *et al*., 2016). IED can incur serious health, social and interpersonal consequences (McLaughlin *et al*., 2012). Although studies in recent decades have provided extensive epidemiological data concerning the mental health of refugees worldwide (Mollica *et al*., 1998; Carlsson *et al*., 2005; Tol *et al*., 2013; Charlson *et al*., 2019; Tay *et al*., 2019*a*), few inquiries have investigated the prevalence and correlates of IED in these populations, a gap in the literature considering the impact of this disorder on refugee families and societies that are already vulnerable because of exposure to past aggression and traumatic events. Persons with IED potentially can cause severe disruptions to these tight-knit communities, which in turn, may exacerbate the ongoing conditions of stress, disruption and deprivation amongst refugee populations. From a public health perspective, it therefore makes sense to gain a better understanding of the problems of explosive anger (represented at the extreme in IED) in defining priorities for psychosocial interventions for refugees. This study aims to examine key aspects of IED amongst three ethnic groups who are refugees from the same country of origin (Myanmar) and relocated to a single country (Malaysia), offering an opportunity to extend our knowledge of the epidemiology and correlates of the disorder amongst forcibly displaced populations.

In relation to the formulation of IED, the disorder has a long history, being included in the first edition of the Diagnostic and Statistical Manual of Mental Disorders (DSM) in 1956 (Coccaro *et al*., 1998). Nevertheless, DSM-5 introduced fundamental changes to the definition of the disorder, specifying more clearly operationalised criteria, that is, the occurrence of episodes of either verbal aggression (tirades, verbal fights) or physical aggression (directed towards property, animals or individuals); a frequency of episodes of occurring at least twice weekly on average over a 3-month period or as less frequent but regular episodes extending over a 12-months (Coccaro *et al*., 1998; Coccaro, 2012; American Psychiatric Association, 2013). The DSM-IV criteria had several limitations, such as not requiring that the aggressive behaviour be impulsive rather than planned (Coccaro *et al*., 2017). This shift from the less stringent DSM-IV (American Psychiatric Association, 2000) criteria is anticipated to reduce the prevalence of IED and hence needs to be taken into account when comparing studies conducted using different definitions over recent decades. Specifically, there is a need to update our understanding of the disorder in relation to its prevalence given that most past studies are based on DSM-IV criteria alone. A comparison of prevalence comparing DSM IV and DSM 5 criteria is a secondary aim of the current paper.

There is now a well-established body of epidemiological studies focusing on IED in the general population. The evidence suggests that (1) exposure to trauma and violence was strongly associated with a greater risk for IED (Fincham *et al*., 2009; Rees *et al*., 2013; Silove *et al*., 2015); (2) IED was found to have a high degree of comorbidity with other common mental disorders (CMDs) such as depression, anxiety and substance use (Kessler *et al*., 2006; Coccaro, 2012; Silove *et al*., 2015), as well as medical conditions (McCloskey *et al*., 2010) and functional impairment (Kessler *et al*., 2006).

Despite the strong evidence of IED in non-refugee populations, there is comparatively limited research in populations exposed to war and mass conflict including refugees and displaced people. IED is particularly relevant to refugee populations because several studies have shown a strong relationship between exposure to prior traumatic events and the disorder (Rees *et al*., 2013; Tay *et al*., 2015*a*). Nevertheless, there are notable anomalies concerning the relationship between trauma and IED in the epidemiological findings of the limited number of studies conducted amongst populations exposed to war and mass conflict. For example, a nationally representative-wide study conducted in Iraq, a country exposed to a succession of wars and conflicts over a protracted period, yielded a lifetime IED prevalence of 1.7% and a 12-month prevalence of 1.5% (Al-Hamzawi *et al*., 2012). These figures are low in comparison to most non-war-affected countries in which IED has been assessed, particularly in high-income countries (Kessler *et al*., 2006; Fincham *et al*., 2009; Coccaro, 2012). There are a range of possible reasons for this finding, including cultural and ethnobiological differences across populations and variations in the case finding measures used. We note that in contrast to the findings in Iraq, our studies, undertaken independent of each other in two different conflict-affected countries (Timor Leste and West Papua), used a different approach to the identification of IED and yielded similarly high IED prevalence estimates of 12% (Tay *et al*., 2015*b*, 2018, 2019*a*).

Consistent with findings amongst civilian populations in high-income countries (Nickerson *et al*., 2012; Scott *et al*., 2016), in the studies amongst West Papuans and Timorese, we identified associations between IED and past exposure to traumatic events related to interpersonal violence, sexual violence and other forms of abuse (Rees *et al*., 2013; Tay *et al*., 2015*b*). In addition, persons with IED reported a high level of exposure to daily stressors. At the same time, some of the sociodemographic factors associated with IED differed from those found in high-income countries, in that women in our studies were at higher risk of IED – which we speculated may have been related to the prevalence of sexual abuse experienced (during episodes of mass conflict and more generally arising from domestic violence). Further, IED was also more prevalent amongst those who were married and the older generation (Rees *et al*., 2013).

There is a growing consensus (Silove *et al*., 2017) that the overall eco-social environment – including cultural, social, economic and environmental factors – shapes the capacity of refugees to adapt to the postmigration environment. The broad eco-social framework encompasses specific traumas and immediate living difficulties in the postmigration environment (Brundige *et al*., 2004; Steel *et al*., 2009) with strong evidence that these contextual stresses influence the mental health impact of discrete traumas (Tay and Silove, 2016; Silove *et al*., 2017). However, the association of postmigration stresses with IED amongst the refugee population from Myanmar are not known. In addition to the contextual stresses, key differences in the history, culture and ethno-biological backgrounds of each ethic group escaping from Myanmar, operating in concert, are likely to account for variations in IED prevalence rates across studies of single populations. Although it remains difficult to differentiate and measure each of these inter-related factors, their composite influence can be broadly assessed by comparing ethnic groups who have lived within their separate cultural contexts in a single country of origin with those who have been similarly forced to migrate to a common country of asylum. These conditions pertain to the several ethnic minorities who have fled Myanmar to neighbouring Malaysia, offering the possibility of examining in a naturalistic manner the effects of ethnocultural group membership to the prevalence and correlates of IED across these distinct communities.

The aims of the present study therefore are to estimates the overall and group-specific prevalence of IED amongst three ethnic groups (Chin, Kachin and Rohingya) from Myanmar living in Kuala Lumpur, Malaysia. To assess the correlates of IED (measured using the DSM5 criteria), specifically the robustness of relationships of exposure to past traumatic events and postmigration living difficulties (PMLDs) with IED (in addition to sociodemographic factors) and to examine the extent to which the prevalence of IED varies according to DSM-IV and DSM5 criteria in these groups.

In addition, we examine patterns of comorbidity of IED with other CMDs (depression, anxiety and PTSD) and the extent to which IED (DSM IV criteria) is associated with functional impairment. We used the identical sampling framework and case-finding method to study the three ethnic groups, adapting measures to the culture and language of each group by applying the same methodology in each instance.

## Method

### Study setting and participants

We derived sample size estimates from health statistics provided by the United Nations High Commissioner for Refugees (UNHCR) who had previously identified the sites of concentration of the three relevant communities living around the Malaysian capital Kuala Lumpur. We applied a cluster, probability, proportional-to-size sampling framework. Based on our power analyses (90% power; alpha = 0.05, two-sided test), we calculated that the minimum acceptable sample size for the Chin was 660, for Kachin 300 and for Rohingya 600 persons. Sampling was undertaken amongst these three communities living in and around Kuala Lumpur between May 2017 and May 2018. Individual households were mapped using the conventional street walk method and, in each dwelling, we identified all adult inhabitants identified as Chin, Kachin or Rohingya or who were offspring of at least one parent from the relevant community. Eligible persons 18-years and older were recruited.

In this manner, three parallel and methodologically identical epidemiological surveys were conducted amongst the Chin, Kachin and Rohingya respectively.

We interviewed a total of 751 Chin (response rate 80%), 338 Kachin (85%) and 959 Rohingya (83%), that is, a combined sample of 2058 participants. The vast majority of eligible residents not interviewed constituted persons who had left the catchment area temporarily for work opportunities in other regions of the country.

### Historical background

More than a million refugees from Myanmar are resettled in Malaysia, the largest country of refuge for displaced populations in the region after Bangladesh. Three of the prominent ethnic groups who have experienced displacement to Malaysia (and to a lesser extent, other Southeast Asian countries) over several decades are the Chin, Kachin and Rohingya.

All three ethnic groups in Malaysia live in tight-knit communities which preserve their distinctive dialects and cultural traditions. All refugees in Malaysia are at risk of discrimination, arbitrary arrest, detention and deportation. Most have no or little access to healthcare, education or secure shelter, and many refugees live under conditions of chronic poverty and food insecurity. The majority work illegally under difficult conditions to support themselves and their families.

### Survey instruments

We used the Refugee Mental Health Assessment Package (R-MHAP) (Schilling *et al*., 2007) to assess sociodemographic characteristics (age, gender, marital status, highest level of educational attainment and employment status), exposures to pre-migration traumatic events (TEs) and post-migration living difficulties (PMLDs) (Tay *et al*., 2019*b*). We included three commonly studied mental disorders (CMDs) in the field i.e. Posttraumatic Stress Disorder (PTSD), Generalized Anxiety Disorder (GAD) ad Major Depressive Disorder (MDD) assessed according criteria of the DSM-5 (American Psychiatric Association, 2013). In addition to this we also added IED diagnostic measures as of the DSM-IV (American Psychiatric Association, 2000) and DSM-5 diagnostic criteria (American Psychiatric Association, 2013).

### Cultural and contextual adaptation of measures

The R-MHAP has undergone extensive development and testing (Schilling *et al*., 2007; IBMCorp, 2019). Consistent with its previous use, we followed a standard protocol to translate the relevant modules into the Burmese and Rohingya languages based on qualitative assessments, in which we refined the items to ensure ease of comprehension, cultural sensitivity and appropriateness and feasibility of application in the relevant populations (Tay *et al*., 2015*b*, 2018, 2019*a*). Serial semantic and linguistic modifications were made in a process of iterative consultation with each ethnic community based on group feedback in which persons of different ages, gender and social status were included. We also consulted two Burmese psychiatrists and two Rohingya-speaking psychologists in the process.

### Pre-migration traumatic events (TEs)

The list of pre-migration TEs was compiled from consultations with all three communities regarding the most common and disturbing conflict-related occurrences experienced in the homeland. Items included exposure to war, torture, persecution, rape, murders, physical injuries, imprisonment, witnessing atrocities and witnessing deaths of family members (each item rated as event occurred = 1; or not occurred = 0). We assessed lifetime exposure (yes = 1, no = 0) based on previous experience which revealed inaccuracies in attempting to obtain fine-grained information regarding the frequency or timing of events in cultures with a low level of numeracy and affinity for historical timing of events. The range of potential trauma scores was 0 to 25. For bivariate and multiple logistic regression analyses, total trauma count was assigned to three hierarchical categories based on the balancing of distributions (0 = 0–9 TEs, 1 = 10–15 TEs, 2 = 16 TEs or greater).

### Post-migration living difficulties (PMLDs)

An inventory of PMLDs (IBMCorp, 2019; Tay *et al*., 2019*c*) was used to assess common stressors confronted by each community in Malaysia based on a locally adapted version of the Humanitarian Emergency Settings Perceived Needs Scale (HESPER). The measure comprised 25 items assessing access to shelter, water, hygiene facilities, employment, education, healthcare and social services. Each of the items was rated on a four-point Likert scale (0 = no problem at all, 1 = a bit of problem, 2 = moderately serious problems and 3 = a very serious problem). We employed two steps in simplifying the scores for analytic purposes (IBMCorp, 2019). First, because the extreme options received low endorsement, we collapsed individual item scores into two categories (0 = not a problem or a bit of a problem; 1 = a moderately or a very serious problem). To facilitate the cross-tabular (bivariate) and multiple logistic regression analysis, we then divided the total PMLD count into three hierarchical categories (0 = 0–9, 1 = 10–15, 2 = 16 and more PMLDs) based on the examination of the distributions of scores. Internal reliability based on Cronbach's alpha (*α*) was high for this measure for all three samples (Chin: *α* = 0.92; Kachin: *α* = 0.96; Rohingya: *α* = 0.88), respectively.

### Common mental disorders (CMDs)

We selected three of the most studied mental health problems in the refugee field that is PTSD, GAD and MDD measured according to DSM-5 diagnostic criteria. Respondents rated the frequency of symptoms on a four-point scale (1 = not at all, 2 = a little bit, 3 = quite a lot, 4 = extremely); endorsement of the two highest frequency categories is recorded as a positive response. Participants completed the full symptom list for each disorder without the application of any skip rules. Responses were recorded directly on an electronic tablet to reduce errors in transcription and ensure rapid access to the data. Past psychometric analyses (Schilling *et al*., 2007) of both categorical and dimensional measures of the three CMDs assessed by the R-MHAP yielded evidence of sound internal consistency, test-retest reliability and concurrent validity of the relevant diagnostic constructs when compared to an established gold standard diagnostic assessment conducted by trained mental health professionals [38]. To determine the prevalence of individual DSM-5 CMDs i.e. PTSD, GAD and MDD, a categorical assignment was made for persons endorsing ‘1’ for DSM-5 threshold criteria satisfied and ‘0’ for not satisfied DSM-5 threshold criteria. In order to determine the prevalence of overall CMDs, firstly we added scores (0, 1) of all three individual CMDs and then we grouped the total number of CMDs as binary categories: ‘0’ for no CMD and ‘1’ for at least one or more CMDs.

### Functional impairment

The World Health Organization Disability Assessment Schedule (WHODAS 2⋅0, 12-item version) has been extensively used across cultures and comprises six core functions/domains relating to cognition/communication, going out (mobility), self-care, interpersonal interactions, life activities (work, home) and participation in society (Von Korff *et al*., 2008; Üstün *et al*., 2010). Ratings for each item range from no impairment = 1 to extreme impairment = 5 (total score range 12 to 60 in the present sample).

### Intermittent explosive disorder (IED)

This study used IED measures as of the DSM-IV (American Psychiatric Association, 2000) and DSM-5 diagnostic criteria (American Psychiatric Association, 2013) to estimate the prevalence of IED for the past 12-month prior to the interview. Respondents were administered the Composite International Diagnostic Interview (CIDI), a fully structured face-to-face interview designed to be administered by trained interviewers.

### Field team and training

The field team consisted of 22 research assistants (12 men and 10 women) drawn from the local Chin (*n* = 7), Kachin (*n* = 5) and Rohingya (*n* = 10) communities. Separate 2-week training for each group which in summary covered basic interviewing techniques, basic counselling, refugee mental health, cultural sensitivity, research ethics, risk assessment and management and referral. Didactic and practical components were included. Sessions commenced with introductory information on refugee mental health, basic mental health concepts and diagnosis and interviewing techniques. Time was devoted to discussing culture-specific aspects of psychiatric presentations, engagement and interviewing, relevant to each ethnic group. Each CMD (PTSD, GAD and MDD) was examined in detail to ensure that all personnel achieved a familiarity with the component symptoms and their variations based on their common experiences and observations and/or that of others. The didactic component of training employed case scenarios, simulated interviews and case formulations. Following the 2-week training, each team then conducted pilot interviews according to the format of the planned survey for a 6-month period in the field, allowing iterative refinement of their approach and the methodology during regular individual and group feedback sessions with supervisors. We required a consistent level of 90 per cent inter-rater reliability (IRR) in diagnostic assignments between trainees and supervisors for the former to be certified to undertake definitive assessments. If workers fell below this level, further training, supervision and field work were conducted; the required level of concordance was achieved. The field team received weekly onsite and remote supervision via videoconferencing throughout the survey. Interviews were conducted at the home of each participant taking on average 45 to 60 min. In relation to the IRR assessments, following 6 months of pilot assessments, all (exceeded 90% pass rate) but two field personnel (on the Rohingya team) achieved 85% on the number of correct case assignment. Further training and 1:1 supervision were provided accordingly and a satisfactory rating was achieved thereafter.

### Statistical analyses

First, we tabulated the descriptive statistics for sociodemographic characteristics (age, gender, employment status, educational attainment, marital status, TEs and PMLDs) and the presence of any CMDs (including the prevalence of PTSD, GAD and MDD respectively). Descriptive statistics were calculated independently for each the three ethnic group (Chin, *n* = 751; Kachin, *n* = 338; and Rohingya, *n* = 959; all from Myanmar) and for the pooled sample (*n* = 2058). We conducted two-way comparisons (that is, Chin *v*. Kachin, Chin *v*. Rohingya and Kachin *v*. Rohingya) to examine for any differences across the groups. We then performed bivariate analyses to examine the association of sociodemographic factors with the prevalence of DSM-IV and DSM-5 IED respectively. The analyses were performed on the pooled sample (*n* = 2058) using the Chin ethnicity as the reference category, considering the low base prevalence rate of IED (3.4% met DSM-5 criteria for IED).

Results of bivariate analyses are presented as percentages and means; statistical tests of significance such as chi-square (*χ*^2^) and *t* tests were applied to examine the significant differences across sub-groups. Through bivariate analyses we also examined the association of DSM-IV and DSM-5 IED with the presence of DSM-5 CMDs (including PTSD, GAD, MDD and overall CMDs respectively). To examine the impact of IED on functional impairment, *t*-tests were conducted to explore the association of diagnostic categorisation (present-absent) and the mean score for each of the six domains (cognition, mobility, self-care, getting along, life activities and community participation) and as well as total functional impairment score respectively. Participants with missing data were excluded from all the analyses based on the assumption that this occurred randomly. All the analyses in this study were performed in SPSS version 26 (IBMCorp, 2019).

## Results

### Descriptive data

The mean age of the combined sample of 2058 persons was 30.5 years (s.d. = 9.3); more than half (53.7%) was aged between 18 and 29 years, 41.6% aged between 30 and 49 years, and the remainder (4.7%) were 50 years and older (Table 1). On average, Rohingya were younger than both the Chin (mean = 28.3 years, vs 32.1 years) and Kachin (mean = 32.9) (both comparisons *p* < 0.001, respectively). Almost two-thirds (65.4%) of the combined sample were male; men were proportionately more highly represented amongst the Rohingya (81.3%) than both the Chin (55.2%) and Kachin (45.4%) (Chin *v*. Kachin: *p* = 0.022; Chin *v*. Rohingya: *p* < 0.001; Kachin *v*. Rohingya: *p* < 0.001). (We note that this pattern of male predominance is characteristic of the whole population of Myanmar refugees in Malaysia). Almost half (48.2%) of Chin and two thirds of the other two ethnic groups (63.5% of Kachin and 69.3% of Rohingya) were employed (Table 1). About 63.2% of all participants either never attended school or had only received some primary school education, 31.5% had completed secondary/high school, and only 5.3% had post-high school or university level education. Higher education levels were low across all three ethnic groups, with only 4.2% of Chin, 14.0% of Kachin and 3.1% of Rohingya completing some form of post-high school education.

**Table 1.** Sociodemographic characteristics and mental health indices of combined total sample (*n* = 2058) and as well three community samples of Chin (*n* = 761), Kachin (*n* = 338) and Rohingya (*n* = 959) refugees living in Malaysia

Characteristics	All *n* (%)	Chin *n* (%)	Kachin *n* (%)	Rohingya *n* (%)	*p*-values: Chin *v*. Kachin	*p*-values: Chin *v*. Rohingya	*p*-values: Kachin *v*. Rohingya
All^a^	2058 (100)	761 (100)	338 (100)	959 (100)			
Age group (in years)							
18–29	1070 (53.7)	344 (45.8)	128 (38.3)	598 (65.9)	0.022	<0.001	<0.001
30–49	829 (41.6)	376(50.1)	186 (55.7)	267 (29.4)	0.087	<0.001	<0.001
50 and above	93 (4.7)	31(4.1)	20 (6.0)	42 (4.6)	0.180	0.617	0.332
Mean (s.d.)	30.5 (9.1)	32.1 (8.6)	32.9 (9.3)	28.3 (9.0)	0.457	<0.001	<0.001
Gender: Female	695 (34.6)	340 (44.8)	184 (54.6)	171 (18.7)	0.003	<0.001	<0.001
Male	1315 (65.4)	419 (55.2)	153 (45.4)	743 (81.3)	0.003	<0.001	<0.001
Employment status							
Unemployed	729 (40.2)	379 (51.6)	119 (36.5)	231 (30.7)	<0.0.01	<0.001	0.062
Employed	1084 (59.8)	356 (48.4)	207 (63.5)	521 (69.3)	<0.001	<0.001	0.062
Educational attainment							
Primary or less	1230 (63.2)	377 (52.8)	54 (16.8)	799 (87.7)	<0.001	<0.001	<0.001
Completed high school	613 (31.5)	307 (43.0)	222 (69.2)	84 (9.2)	<0.001	<0.001	<0.001
University/training	103 (5.3)	30 (4.2)	45 (14.0)	28 (3.1)	<0.001	<0.001	0.222
Marital status							
Single/unmarried	1122 (64.5)	80 (12.4)	128 (40.8)	360 (43.6)	<0.001	<0.001	0.389
Married/partnered	94 (4.6)	555 (86.2)	163 (51.9)	404 (48.9)	<0.001	<0.001	0.362
Divorced/Widowed	842 (40.9)	9 (1.4)	23 (7.3)	62 (7.5)	<0.001	<0.001	0.920
Number of total TEs							
None	71 (3.4)	9 (1.2)	0 (0.0)	62 (6.5)			
1 to 9	975 (47.4)	518 (68.1)	146 (43.2)	311 (32.4)	<0.001	<0.001	0.164
10 to 15	667 (32.4)	163 (21.4)	136 (40.2)	368 (38.4)	<0.001	<0.001	0.548
16 or more	218 (16.8)	71 (9.3)	56 (16.6)	218 (22.7)	0.001	<0.001	0.0168
Mean (s.d.)	9.7 (9.0)	8.2 (5.2)	11.5 (5.0)	11.1 (6.4)	<0.001	<0.001	0.656
Number of PMLDs							
None	169 (8.2)	99 (13.0)	70 (20.7)	0			
1 to 9	768 (37.3)	407 (53.5)	126 (37.3)	235 (24.5)	0.006	<0.001	<0.001
10 to 15	548 (26.6)	137 (18.0)	49 (14.5)	362 (37.7)	0.152	<0.001	<0.001
16 or more	573 (27.8)	118 (15.5)	93 (27.5)	362 (37.7)	<0.001	<0.001	0.001
Mean (s.d.)	7.8 (7.1)	7.4 (6.6)	8.7 (8.0)	13.5 (5.4)	0.006	<0.001	<0.001
DSM-5 PTSD criteria met: Yes	452 (22.0)	5 (0.7)	18 (5.3)	429 (44.7)	<0.001	<0.001	<0.001
DSM-5 GAD criteria met: Yes	809 (39.3)	146 (19.2)	85 (25.1)	578 (60.3)	0.025	<0.001	<0.001
DSM-5 MDD criteria met: Yes	848 (41.2)	170 (22.3)	102 (30.2)	576 (60.1)	0.005	<0.001	<0.001
Number of CMDs^b^							
None	932 (45.3)	536 (70.4)	213 (63.0)	183 (19.1)	0.015	<0.001	<0.001
One or more CMDs	1126 (54.7)	225 (29.6)	125 (37.0)	776 (80.9)	0.015	<0.001	<0.001
DSM-4 IED criteria met: Yes	122 (5.9)	24 (3.2)	31 (9.2)	67 (7.0)	<0.001	<0.001	0.190
DSM-5 IED criteria met: Yes	71 (3.4)	16 (2.1)	27 (8.0)	28 (2.9)	<0.001	0.284	<0.001

a *Notes:* The numbers do not always add up to exact total due to exclusion of missing/inadequately described’ data.

b Number of CMDs are calculated based on combination PTSD, GAD and MDD.

Abbreviations: TEs, Pre-migration traumatic exposures; PMLDs, Post-migration living difficulties; DSM-5, Diagnostic and Statistical Manual for Mental Disorder (5th revision); PTSD, Post-traumatic stress disorder; MDD, Major Depressive Disorder; GAD, Generalised anxiety disorder; CMDs, common mental disorders; IED, Intermittent Explosive Disorder, s.d., Standard deviation.

Almost two-thirds (64.5%) of participants were married/partnered, 40.9% were single or never married and the remaining 4.6% were divorced/widowed or separated (Table 1). A higher proportion of Chin were married (86.2%) compared to both the Kachin (51.9%) and Rohingya (48.9%).

The mean number of premigration TEs was 9.7 (s.d. = 6.4); 96% reported experiencing at least one TE and about half reported 10 or more of these events. More than half of the Kachin (58.6%) and Rohingya (61.1%) reported 10 or more exposures to premigration TEs, compared to 30.7% of the Chin (*p* < 0.001 for comparisons between Kachin and Chin and between Rohingya and Chin, respectively).

The mean number of PMLDs endorsed was 7.8 (s.d. = 7.1), with more than half of the participants reporting 10 or more of these stressors at a high level (Table 1). Rohingya refugees reported higher PMLDs (mean = 13.5, s.d. = 5.4) compared to the Chin (mean = 7.4, s.d. = 6.6) and Kachin (mean = 8.7, s.d. = 8.0) (both comparisons *p* < 0.001). Almost three-quarters of Rohingya (75.4%) reported 10 or more PMLDs, compared to 42.0% of Kachin and 33.5% of the Chin (Table 1).

Of the total pool of participants, 22.2% met DSM-5 criteria for PTSD, 39.3% GAD and 41.2% MDD. Overall, more than half (54.7%) of all participants met the criteria for at least one of the measured CMDs (Table 1). Rohingya recorded the highest prevalence of all three CMDs (80.9% had at least one CMD), compared to the Kachin (37.0%) and Chin (29.6%) (all two-way group differences *p* < 0.01). Over half of the Rohingya (56.2%) had two or more CMDs compared to 12.3% for the Chin and 19.8% for the Kachin (Table 1).

### DSM-IV and DSM-5 IED and associations with sociodemographic factors

5.9% (122 out of 2058) of all participants met 12-month IED criteria for DSM-IV and 3.4% (71 out of 2058) DSM-5 12-month IED criteria. Kachin had the highest IED prevalence, followed by Rohingya and Chin (Table 2).

**Table 2.** Prevalence of DSM-4 IED and DSM-5 IED by Sociodemographic characteristics for Myanmar Refugees Living in Malaysia (*n* = 2058)

Sociodemographic characteristics	Total participants	Prevalence of DSM-4 IED		Prevalence of DSM-5 IED	
	Number	Number	Prevalence (row %)	Number	Prevalence (row %)
**All**	**2058**	**122**	**5**.**9**	**71**	**3**.**4**
Name of Refugee Community					
Chin	761	24	3.2	16	2.1
Kachin	338	31	9.2	27	8.0
Rohingya	959	67	7.0	28	2.9
*p-values from Chi-square test*			*p* = 0.001		*p* = 0.001
Gender					
Female	695	29	4.2	22	3.2
Male	1315	93	7.1	49	3.7
*p-values from Chi-square test*			*p* = 0.002		*p* *=* *0.517*
Age group (in years)					
Under 30	1070	73	6.8	40	3.7
30–49	829	47	5.7	29	3.5
50 and above	93	1	1.1	1	1.1
*p-values from Chi-square test*			*p* *=* *0.069*		*p* *=* *0.408*
Employment					
Unemployed	729	45	6.2	30	4.1
Employed	1084	60	5.5	29	2.7
*p-values from Chi-square test*			*p* *=* *0.569*		*p* *=* *0.090*
Educational attainment					
Primary or less	1230	86	7.0	46	3.7
Completed high school	613	30	4.9	21	3.4
University/training	103	3	2.9	2	1.9
*p-values from Chi-square test*			*p* *=* *0.079*		*p* *=* *0.626*
Marital status					
Married/widowed/separated	1216	49	4.0	22	1.8
Single/unmarried	842	73	8.7	49	5.8
*p-values from Chi-square test*			*p* = 0.002		*p* = 0.004
Number of TEs exposures					
0 to 9	1046	32	3.1	19	1.8
10 to 15	667	46	6.9	29	4.3
16 or more	345	44	12.8	23	6.7
*p-values from Chi-square test*			*p* = 0.002		*p* = 0.001
Number of PMLDs					
0 to 9	937	26	2.8	12	1.3
10 to 15	548	38	6.9	20	3.6
16 or more	573	58	10.1	39	6.8
*p-values from Chi-square test*			*p* = 0.003		*p* = 0.002

Abbreviations: TEs, Pre-migration traumatic exposures; PMLDs, Post-migration living difficulties; IED, Intermittent Explosive Disorder; DSM-4, Diagnostic and Statistical Manual for Mental Disorder (4th revision); DSM-5, Diagnostic and Statistical Manual for Mental Disorder (5th revision).

P=0.001

Bivariate analyses supported a generic pattern of associations between IED and key correlates. Single status was associated with both DSM-IV and DSM-5 IED in all ethnic groups (all *p* < 0.01); and a similar pattern was found for premigration TEs experienced (all *p* < 0.001) and number of PMLDs (all *p* < 0.001). A gender difference (higher prevalence of IED for males) was only evident for DSM-IV (*p* < 0.05), but not DSM5 IED.

A closer examination of TEs showed a consistent linear relationship of this variable with both DSM-IV and DSM-5 IED (Table 2). For DSM-IV, IED prevalence rose from 3.1% for 0–9 TEs to 12.8% for 16 or more TEs. DSM-5 IED prevalence rose from 1.8% for 0–9 TEs to 6.7% for 16 or more TEs. Number of PMLDs showed a similar linear pattern of association: DSM-IV IED prevalence rose from 2.8% for 0–9 PMLDs to 10.1% for 16 or more PMLDS; DSM-5 IED prevalence rose from 1.3% for 0–9 PMLDs to 6.8% for 16 or more PMLDs.

### Association of DSM-IV and DSM-5 IED with CMDs

Participants from the whole sample who met either DSM-IV or DSM-5 IED criteria had significantly higher rates of PTSD, GAD and MDD as compared those who did not meet IED criteria in either classification (Table 3). Based on DSM-5 defined symptom counts, IED was positively correlated with CMDs including PTSD (*r* = 0.43, *p* < 0.001); GAD (*r* = 0.78, *p* < 0.001) and MDD (*r* = 0.73, *p* < 0.001) respectively. From a categorical diagnostic perspective, of those participants who met DSM5 criteria for at least one other CMD, 85.2% met DSM-IV IED criteria and 81.7% DSM-5 IED criteria.

**Table 3.** Presence of IED by DSM-5 common mental health disorders (CMDs) for Myanmar Refugees Living in Malaysia

	Total participants	Presence of DSM-4 IED			Presence of DSM-5 IED		
DSM-5 Mental health indices		No IED	With IED	*p-values*	No IED	With IED	*p-values*
	Number (col %)	Number (col %)	Number (col %)		Number (col %)	Number (col %)	
All	2058 (100)	1936 (100)	122 (100)		1987 (100)	71 (100)	
DSM-5 PSTD criteria met							
No	1606 (78.0)	1539 (79.5)	67 (54.9)		1565 (78.8)	41 (57.7)	
Yes	452 (22.0)	397 (20.5)	55 (45.1)	*p* = 0.002	422 (21.2)	30 (42.3)	*p* = 0.002
DSM-5 GAD criteria met							
No	1249 (60.7)	1211 (62.6)	38 (31.1)		1227 (61.8)	22 (31.0)	
Yes	809 (39.3)	725 (37.4)	84 (68.9)	*p* = 0.002	760 (38.2)	49 (69.0)	*p* = 0.003
DSM-5 MDD criteria met							
No	1210 (58.8)	1172 (60.5)	38 (31.1)		1187 (59.7)	23 (32.4)	
Yes	848 (41.2)	764 (39.5)	84 (68.9)	*p* = 0.003	800 (40.3)	48 (67.6)	*p* = 0.002
Met criteria for overall CMDs							
No CMDs	932 (45.3)	914 (47.2)	18 (14.8)		919 (46.3)	13 (18.3)	
*One or more CMDs*	1126 (54.7)	1022 (52.8)	104 (85.2)	*p* = 0.001	1068 (53.7)	58 (81.7)	*p* = 0.002

Abbreviations: DSM-5, Diagnostic and Statistical Manual for Mental Disorder (5th revision); PTSD, Post-traumatic stress disorder; GAD, Generalised anxiety disorder; MDD, Major Depressive Disorder; CMDs, common mental disorders; IED, Intermittent Explosive Disorder.

### Association of DSM-IV and DSM-5 IED with functional impairment

Total WHODAS functional impairment score was significantly higher for participants who met either DSM-IV IED criteria (no IED: mean = 17.1, s.d. = 10.5; IED present: mean = 22.2, s.d. = 11.4; *p* < 0.001) and DSM-5 criteria (no IED: mean = 17.3, s.d. = 10.6; presence of IED: mean = 21.4, s.d. = 11.4; *p* < 0.001). The same pattern of association was found between each of the WHODAS subdomains and DSM-IV and DSM-5 IED (all <0.05), with the exception of the single domain of mobility (Table 4).

**Table 4.** Associations of intermittent explosive disorder (IED) and functioning according to the WHODAS and its subdomains among Myanmar refugees living in Malaysia (*n* = 2058)

WHODAS Six domains of functional impairment	Total participants	Presence of DSM-4 IED			Presence of DSM-5 IED		
		No IED	With IED	*No IED v*. *IED: p-values from t-test*	No IED	With IED	*No IED v*. *IED: p-values from t-test*
	(*n* = 2058)	(*n* = 1936)	(*n* = 122)		(*n* = 1987)	(*n* = 71)	
	Mean (s.d.)	Mean (s.d.)	Mean (s.d.)		Mean (s.d.)	Mean (s.d.)	
Cognition (understanding & communicating)	3.0 (1.9)	3.0 (1.9)	3.8 (2.3)	*0*.*001*	3.0 (1.9)	3.8 (2.4)	*0*.*005*
Mobility (moving & getting around)	2.9 (2.0)	2.8 (2.0)	3.2 (2.0)	*0*.*052*	2.8 (2.0)	3.0 (2.1)	*0*.*551*
Self-care (hygiene/dressing/eating/staying alone)	2.5 (2.1)	2.5 (2.1)	3.4 (2.7)	*0*.*001*	2.5 (2.1)	3.0 (2.5)	*0*.*043*
Getting along (interacting with other people)	2.7 (2.1)	2.7 (2.0)	3.6 (2.6)	*0*.*001*	2.7 (2.0)	3.4 (2.4)	*0*.*023*
Life activities (domestic responsibilities/leisure)	3.0 (1.9)	3.0 (1.9)	3.5 (1.9)	*0*.*004*	3.0 (1.9)	3.5 (2.1)	*0*.*027*
Participation (joining in community activities)	3.3 (2.0)	3.2 (1.9)	4.7 (1.9)	*0*.*001*	3.2 (2.0)	4.7 (2.4)	*0*.*001*
*All domains of functional impairment*	*17.4* (*10.7)*	*17.1* (*10.5)*	*22.2* (*11.4)*	*p* = 0.002	*17.3* (*10.6)*	*21.4* (*11.4)*	*p* = 0.003

## Discussion

Our study is unique in applying the same methodology to study IED using both DSM-IV and DSM5 criterion in parallel studies of three refugee ethnic groups from the same country (Myanmar) resettled in a common environment (Malaysia).

The 12-month pooled prevalence based on the DSM-5 criteria for IED was 3.4% with a higher prevalence of 5.9% applying to DSM-IV criteria, although there was a notable difference in prevalence across the three ethnic groups. For the three ethnic groups, the prevalence rates for DSM-5 IED and DSM-4 IED were: Chin (2.1% *v*. 3.2%), Kachin (8% *v*. 9.2%), Rohingya (2.9% *v*. 7%). The putative risk factors identified in those who met the criteria for IED included gender (being male but only for DSM-IV criteria), being single, exposure to premigration traumatic events and to later post-migration living difficulties. Furthermore, our findings showed a significant dose-response pattern for both premigration traumatic events and PMLDs with IED prevalence, and high levels of comorbidity and functional impairment with IED prevalence.

Prior to discussing the implications of our findings, we note the strengths and limitations of our study. The strengths included the use of the same probabilistic sampling frame and measures across the three refugee groups; a parallel process of cultural and contextual adaptation; a comparable process of training community field workers from each ethnic group; the successful recruitment of a large sample relative to studies within the refugee field; and a high response rate (in excess of 80% across three ethnic groups). The gender imbalance in the sample, with more men (65.4%) being represented, is consistent with the known profile of the resettled groups in Myanmar.

Limitations of the study are the risk of transcultural errors in the process of adapting measures based on comprehension and potential response biases in endorsing items. Moreover, concurrent validity testing (comparing results obtained by field personnel with diagnoses made by professionals) would provide greater assurance of the accuracy of IED diagnoses.

The cross-sectional design of the study precludes any firm inferences being drawn about causal connections between putative risk factors and outcomes. Retrospective bias in recall of trauma events may introduce inaccuracies in reporting. We note, however, that the reported pattern of exposure to traumatic events is consistent with the known history of each refugee group displaced from Myanmar to Malaysia.

In relation to the main findings, it is notable that the prevalence of IED identified in the combined sample appeared to be broadly commensurate with the 2.6% to 4.3% range identified amongst international studies based on heterogeneous populations and applying DSM5 criteria (Coccaro *et al*., 2017; Coccaro, 2019). Nevertheless, there was a large variation in prevalence across the three ethnic groups, the notable distinction being the figure for Rohingya (8%) compared with Chin (2.1%) and Kachin (2.9%), although the order of change differed for DSM-IV. This pattern might be partially attributable to the extreme types of mass trauma (e.g., indiscriminate assault, mass killings, sexual violence, witnessing atrocities) of a genocidal nature that the Rohingya had experienced (and their kinship group continued to experience back at home during the time of the survey).

There were notable commonalities across the three groups in the sociodemographic and contextual factors associated with IED across the three groups, in keeping with hypothesised factors of relevance in generating IED. These included family and social isolation (being single), exposure to high levels of premigration traumatic events and to PMLDs. However, we did not replicate the same pattern of socio-demographic factors as other cross-national surveys (Scott *et al*., 2016), which is likely to reflect the particularities of each refugee population in which the persons most affected by the conflict or most likely to migrate may differ in sociodemographic characteristics (e.g., single men) depending on the context.

The consistent finding of our study with the results of past studies amongst culturally diverse refugees and displaced populations is that there was a linear increase in IED prevalence with higher exposure to both past traumatic events and PMLDs, suggesting that these are robust risk factors for the disorder in these populations (Fincham *et al*., 2009; Rees *et al*., 2013; Silove *et al*., 2015; Scott *et al*., 2016). Our finding supports both a theoretical understanding of the relevance of recurrent trauma and stress in the genesis of IED, and the psychosocial factors that may assist in the identification of those at risk.

As has been found universally, IED is highly comorbid with other CMDs (in this case with the MDD, GAD and PTSD) in our sample (Kessler *et al*., 2006; Coccaro, 2012; Scott *et al*., 2016). This suggests that there may be generic pathways to comorbidity amongst populations exposed to traumatic events and posttraumatic stressors leading to multiple comorbid outcomes involving the co-occurrence of CMDs. These observations are consistent with the contemporary perspective of implementing a multi-focused approach to interventions to address comorbid response patterns across a range of trauma-exposed populations. For example, amongst combat veterans, war-affected populations) (Coccaro *et al*., 2004; Kessler *et al*., 2006). Directionality in the pathogenesis of comorbid conditions can be difficult to determine, particularly in cross-sectional studies of this kind, but it is plausible that the presence of IED can lead a multiplicity of personal, familial and social complications which generates more symptoms of depression and anxiety in the individual (Kessler *et al*., 2006). This may be particularly true in refugee populations in which the presence of IED in an individual is likely to have cascade of effects on the cohesion and fabric of tight-knit, traditional communities, resulting in depression and other comorbid complications in the sufferer. Longitudinal studies are necessary to trace the temporal relationships and pathways involving the pathogenesis of IED and its effects on the onset of other CMDs in refugee communities.

From a clinical perspective, our findings support a focus on developing and implementing multipronged psychosocial strategies for identification and interventions for IED and comorbid CMDs amongst refugees. Although several cognitive behavioural therapies have shown promise in treating maladaptive anger and IED in trauma-affected individuals (Litz *et al*., 2009; Borges *et al*., 2020), evidence for effective psychosocial interventions specific for refugees and conflict-affected populations remains limited. A small, trauma-focused psychotherapy formulated specifically to address IED and PTSD amongst conflict-affected person in Timor-Leste has shown promising outcomes (Hewage *et al*., 2018). More recently, a randomised controlled trial of an intervention (Integrative ADAPT Therapy or IAT) based on a psychosocial model specific to refugees conducted amongst the three communities included in the present study recorded positive outcomes for a range of comorbid disorders including IED (Tay *et al*., 2020). An implementation study using a group-based IAT approach amongst Rohingya refugees in Bangladesh provided added evidence in support of the effectiveness of this approach (Tay *et al*., 2021).

## Conclusions

To our knowledge, this is the first study to examine the prevalence and correlates of IED across multiple refugee populations from the one country of origin resettled in the same environment. In the pooled sample, the prevalence of IED was comparable to global trends, but there was substantial variation across the three study groups, a pattern that was partly attributable to exposure to extreme premigration trauma and postmigration stresses as well as other possible ethnocultural differences. A consistent pattern of putative risk factors was identified, that is, being male, single, exposed to a higher number of premigration traumatic events and greater level of PMLDs. Variations in these risk factors are likely to be instrumental in explaining differences in prevalence rates of IED across other refugee groups and more widely, trauma-affected populations. In that sense, our study may have wider implications in understanding the pathogenesis and pattern of expression of IED across populations worldwide.

## Data Availability

The data that support the findings of this study are available from the corresponding author, A. K. T., upon reasonable request.
